# Supplementary material for: Air Pollution and Respiratory System Responses in Healthy Adults Engaging in Outdoor Physical Exercise in Urban Environments: A Scoping Review
Source: Int J Environ Res Public Health. 2025 Aug 28;22(9):1347. doi: 10.3390/ijerph22091347 (PMC12469555; doi:10.3390/ijerph22091347)
Supplement: Supplementary file 1 [file ijerph-22-01347-s001.zip › ijerph-3640206-supplementary.pdf]

Supplementary Material

To promote transparency and reproducibility in the research, the following appendices present essential supplementary materials

Table S1. Search Strategy—21 October 2024 (last search).

Search Equation	Database	Filters	Results
"Air pollution" AND "exercise" AND "respiratory system" AND "outdoors"	Pubmed	No filters<br>Publication year: 2004-2024<br>Language: English, Spanish	**3**
	Redalyc	No filters	**1**
	Scielo	No filters	1 265 416
		Language: English, Spanish	334 631
		Publication year: 2004-2024	273 910
		SciELO subject areas: Health Sciences – Biological Sciences	101 354
		WoS thematic categories: Respiratory	822
		Literature type: Review Article – Original Article – Case Report	**400**
	Web of Science	No filters	**0**
"Air pollution" AND "exercise" AND "outdoors"	Pubmed (Medline)	No filters	48
		Publication year: 2004-2024<br>Disponibilidad: Texto completo	39
		Article types: Adaptive Clinical Trial, Case Reports, Clinical Trial, Controlled Clinical Trial, Meta-Analysis, Randomized Controlled Trial, Review, Systematic Review<br>Language: English, Spanish<br>Especie: Humanos	10
		Age group: Adults 19+ years – Seniors 65+ years<br>Exclude: Preprints	**5**
	Redalyc	No filters<br>Publication year: 2004-2024<br>Language: English, Spanish	12
		Discipline: Health – Medicine	**6**
	Scielo	No filters	1 265 416
		Language: English, Spanish	334 631
		Publication year: 2004-2024	273 910
		SciELO subject areas: Health Sciences – Biological Sciences	101 354
		WoS thematic categories: Respiratory	822
		Literature type: Review Article – Original Article – Case Report	**400**
	Web of Science	No filters	**0**
**Total articles found**			**815**

Source: Own elaboration.

Table S2. Summary of results of methodological quality assessment of included studies—Randomized controlled trial.

**Study**<br>**JBI Checklist for Randomized Controlled Trials**	**Sinharay R, et al.**	**Matt F, et al.**	**Kocot K, Zejda JE**
Was true randomization used for assignment of participants to treatment groups?	Yes	No	No
Was allocation to treatment groups concealed?	Yes	No	No
Were treatment groups similar at baseline?	Yes	Yes	Yes
Were participants blind to treatment assignment?	Yes	Yes	Yes
Were those delivering treatment blind to treatment assignment?	Unclear	No	Yes

Were outcome assessors blind to treatment assignment?	Unclear	No	Yes
Were treatment groups treated identically other than the intervention of interest?	Yes	Yes	Yes
Was follow-up complete, and if not, were differences between groups in terms of follow-up adequately described and analyzed?	Yes	Yes	Yes
Were participants analyzed in the groups to which they were randomized?	Yes	Yes	Yes
Were outcomes measured in the same way for treatment groups?	Yes	Yes	Yes
Were outcomes measured reliably?	Yes	Yes	Yes
Was appropriate statistical analysis used?	Yes	Yes	Yes
Was the trial design appropriate, and were any deviations from the standard RCT design (individual randomization, parallel groups) accounted for in the conduct and analysis of the trial?	Yes	Unclear	Yes
**Percentage of positive responses**	**84.6%**	**61.5%**	**84.6%**

Source: Authors' own elaboration.

Table S3. Summary of methodological quality assessment results of included studies—Quasi-Experimental Studies.

**Study**<br>**JBI Checklist for Quasi-Experimental Studies**	**Pagani LG, et al**	**Strak M, et al.**	**Kocot, K., et al.**
Is it clear in the study what is the "cause" and what is the "effect" (i.e., there is no confusion about which variable comes first)?	Yes	Yes	Yes
Were the participants included in any comparison similar?	Yes	Yes	Yes
Were the participants in any comparison receiving similar treatment or care, other than the exposure or intervention of interest?	Yes	Yes	Yes
Was there a control group?	No	No	No
Were there multiple measurements of the outcome both before and after the intervention/exposure?	Yes	No	Yes
Was follow-up complete, and if not, were differences between groups in terms of follow-up adequately described and analyzed?	Yes	Yes	Yes
Were the outcomes of participants included in any comparison measured in the same way?	Yes	Yes	Yes
Were the outcomes measured reliably?	Yes	Yes	Yes
Was appropriate statistical analysis used?	Yes	Yes	Yes
**Percentage of positive responses**	**88.8%**	**77.7%**	**88.8%**

Source: Authors' own elaboration.

Table S4. Summary of methodological quality assessment results of included studies – Analytical Cross-Sectional Studies.

**Study**<br>**JBI Checklist for Analytical Cross-Sectional Studies**	**Kesavachandran CN, et al.**	**Marmett B**
Were the inclusion criteria in the sample clearly defined?	Sí	Sí
Were the study subjects and the setting described in detail?	Sí	Sí
Was the exposure measured in a valid and reliable way?	Sí	Sí
Were objective, standard criteria used for the measurement of the condition?	Sí	Sí
Were confounding factors identified?	Sí	No es claro
Were strategies to deal with confounding factors stated?	Sí	No es claro
Were the outcomes measured in a valid and reliable way?	Sí	Sí
Was appropriate statistical analysis used?	Sí	Sí
**Percentage of positive responses**	**100%**	**75%**

Source: Authors' own elaboration.

Table S5. Detailed Synthesis of Results.

Author and Publication year	Reported Air Pollutants	Air Quality Measurement Method	Health/Respiratory Assessment Method
Sinharay R, et al. - 2018 [26]	**Approximate average values for Low Pollution/High Pollution:**<br>BC: 0.2 μg/m³ / 11 μg/m³<br>NO_2: 20 ppb / 99 ppb<br>PM_{10}: 18 μg/m³ / 26 μg/m³<br>$PM_{2.5}$: 7 μg/m³ / 18 μg/m³<br>UP: 6000 particles/cm³ / 26000 s/cm³	***$PM_{2.5}$ and PM_{10}:** Light-scattering sensor (AM510 SidePak Personal Aerosol Monitors, TSI Ltd, MI, USA).*<br>***UP:** Unipolar diffusion charger (Philips Aerosense NanoTracer; particle size range: 10 to 300 nm).*<br>***BC:** Optical absorption method (microAeth Model AE51 Black Carbon aerosol monitor; AEthLabs, CA, USA; flow rate of 100 mL per minute).*<br>***T and RH:** Electronic data logger (unclear specifications).*<br>***NO_2:** Fixed monitoring site on Oxford Street. No monitoring available in Hyde Park; data was taken from the nearest representative location.*	**Dyspnea:** Modified Medical Research Council (mMRC) Dyspnea Scale.<br>**Respiratory symptoms** (cough, sputum, wheezing): Rated on a scale from 0 to 4.<br>**FEV1 and FVC:** Spirometry (Vitalograph, Buckingham, UK).<br>**Airway resistance at 5 and 20 Hz (R5 and R20):** Impulse oscillometry (Master Screen Spirometry-IOS System, Jaeger, Germany).<br>**FeNO:** Portable electrochemical sensor (NOBreath, Bedfont Scientific Ltd, Kent, UK).<br>**Pulse wave velocity and augmentation index:** Vicorder device (Skidmore Medical, Bristol, UK).
Matt F, et al. - 2016 [27]	**Average values (Low TRAP/High TRAP):**<br>**NOx:** 102 ppb / 685 ppb<br>**NO:** 77 ppb / 593 ppb<br>**BC:** 6.9 μg/m³ / 28.9 μg/m³<br>**UP:** 45,992 particles/cm³ / 164,708 particles/cm³<br>**PM2.5:** 39 μg/m³ / 82 μg/m³<br>**PM10:** 65 μg/m³ / 123 μg/m³<br>**Coarse PM:** 27 μg/m³ / 41 μg/m³	**UP:** Condensation Particle Counter (CPC, model 3007, TSI, Minnesota, USA).<br>**$PM_{2.5}$ and PM_{10}:** DustTrak (DRX, model 8534, TSI, Minnesota, USA).<br>**BC:** Portable Aethalometer (model AE-51, McAgee Scientific, California, USA).<br>**NO and NOx:** Nitric oxide monitor (model 410, 2B Technologies, Colorado, USA) combined with NO_2 converter (model 401, 2B Technologies, Colorado, USA).<br>**T and RH:** Q-Track (model 7565, TSI, Minnesota, USA) and meteorological station (model WMR80, Oregon Scientific, Buckinghamshire, UK).	***Cardiorespiratory performance:** Finger pulse oximeter (Konica Minolta, Japan)*<br>***Heart rate (HR):** Ambulatory electrocardiography monitor (ModelCardioLight, Gem-Med, Spain)*<br>***Pulmonary function:** Portable spirometer EasyOne (Ndd Medical, Switzerland)*
Kocot K, Zejda JE -2020 [28]	***Exposure Test / Control Test:***<br>***SO_2:** 28.0 ± 18.5 μg/m³ / 9.2 ± 4.5 μg/m³*<br>***NO_x:** 139.6 ± 86.9 μg/m³ / 38.6 ± 23.7 μg/m³*	***PM_{10}:** SidePak AM520 device (TSI, USA).*<br>***$PM_{2.5}$, SO_2 and NO_x:** Monitoring station of the General Inspectorate of Environmental Protection.*	***Blood pressure:** OMRON M2 Basic device (Omron Healthcare, Netherlands).*<br>***SpO_2:** Pulsox-2 oximeter (Konica Minolta, Japan).*<br>***Spirometry:** Easy One spirometer*

	PM_{10}: *189.3 ± 121.3 μg/m³ / 32.9 ± 28.1 μg/m³* ***$PM_{2.5}$:*** *75.3 ± 36.2 μg/m³ / 26.1 ± 15.6 μg/m³*		*(NDD, Switzerland).* ***FeNO:*** *NIOX Mino (Aerocrine AB, Sweden) and Vivatmo Pro device (Bosch, Germany).*
Pagani LG, et al.-2020 [29]	**Particle levels 10 weeks before baseline / after baseline:** **$PM_{2.5}$:** 17.26 ± 13.75 μg/m³ / 21.69 ± 20.69 μg/m³ **PM_{10}:** 29.24 ± 19.26 μg/m³ / 37.32 ± 23.83 μg/m³	CETESB database.	***Aerobic capacity:*** *Ellestad protocol.* ***Nasal lavage IL concentrations (IL-6, IL-10, IL-17A, TNF-α):*** *U-PLEX MSD multiplex kit (Meso Scale, Discovery, Rockville, MD, USA).* ***Protein concentration in nasal lavage:*** *BCA kit developed by Pierce™ (Pierce Biotechnology, Rockford, IL, USA).* ***Nasal NO:*** *Chemiluminescence detection using Sievers NO analyzer (model Sievers NOA 280, Boulder, CO, USA).*
Strak M, et al.-2010 [30]	**Median concentrations on Low Traffic / High Traffic routes**: **PM_{10}:** 45.44 μg/m³ / 37.34 μg/m³ **BC:** 3.79×10⁻⁵/m / 5.50×10⁻⁵/m **PNC:** 27,028 particles/cm³ / 41,097 particles/cm³	**PNC (Concentración numérica de partículas):** Contador de partículas de condensación en tiempo real (CPC modelo 3007; TSI, St. Paul, MN, EE. UU.). **PM_{10}:** Impactadores Harvard con dos bombas de interior (modelo SP-280E; Air Diagnostics and Engineering, Harrison, ME, EE. UU.). **Recolección de partículas:** Filtros de teflón (37 mm de diámetro, 2 μm de tamaño de poro, anillo de soporte de PVC; Pall, Port Washington, NY, EE. UU.). **Masa de las partículas:** Microbalanza Mettler MT5 (Mettler-Toledo, Greifensee, Suiza). **BC (Soot):** Smoke stain reflectometer (model M43D; Diffusion Systems, London, UK). **Temperature and relative humidity:** Oakton RH/TempLog data logger (Oakton Instruments, Vernon Hills, Illinois, USA).	**Lung function and FeNO (fractional exhaled nitric oxide):** Assessed using a respiratory symptom questionnaire. **Lung function (spirometry):** Measured using a portable electronic spirometer, Micro Medical Diary Card (Micro Medical, Rochester, UK). **FeNO:** Measured with the Niox Mino monitor (Aerocrine, Solna, Sweden).
Kocot, K., et al. – 2021 [31]	**Average Recorded Values – Intervention / Control:** **Indoor $PM_{2.5}$**: 114 μg/m³ / 27 μg/m³	***Indoor PM2.5 and PM10:*** *SidePak AM520 (TSI, Shoreview, MN, USA).* ***Outdoor SO_2 and NO_2:*** *Mobile laboratory – T100 and T200 analyzers*	***Lung function:*** *Spirometry performed using the Easy One Air device (NDD, Zurich, Switzerland).*

	**Indoor PM_{10}**: 155.2 μg/m³ / 45.3 μg/m³<br>**Outdoor PM_{10}**: 127.3 μg/m³ / 33.4 μg/m³<br>**Outdoor SO_2:** 26.2 μg/m³ / 28.3 μg/m³<br>**NO_2:** 50.6 μg/m³ / 29.3 μg/m³	*(Teledyne Advanced Pollution Instrumentation, San Diego, CA, USA).*<br>***Outdoor PM:*** *BAM1020 (Met One Instruments, Grants Pass, OR, USA) – Weather conditions monitored with WS500 (Lufft, Fellbach, Germany).*	***Blood pressure:*** *Not clearly specified.*<br>***FeNO:*** *Not clearly specified.*
Kesavachandran CN, et al.- 2015 [32]	**Overall Average Values:**<br>• **$PM_{2.5}$:** 241.5 μg/m³<br>• **PM_{10}:** 121.2 μg/m³<br>**Minimum Values:**<br>• **$PM_{2.5}$:** 132 μg/m³<br>• **PM_{10}:** 12 μg/m³<br>**Maximum Values:**<br>• **$PM_{2.5}$:** 323 μg/m³<br>• **PM_{10}:** 246 μg/m³<br>•	***PM:*** *Automated real-time instrument for ambient air monitoring, HAZ-DUST (EPAM-5000, Environmental Devices Corporation, USA).*	***Heart rate (HR):*** *Manual measurement of radial pulse.*<br>***Self-reported respiratory illnesses:*** *Questionnaire-based assessment.*<br>***PEF and FEV_1:*** *Dry spirometer (PIKO, UK).*
Marmett B, et. al.- 2023 [33]	***Maximum O_3:*** *52.63 μg/m³ (8-hour average)*<br>***Minimum O_3:*** *7.15 μg/m³ (8-hour average)*<br>***Maximum NO_2:*** *22.54 μg/m³ (24-hour average)*<br>***Minimum NO_2:*** *18.78 μg/m³ (24-hour average)*	**O_3 and NO_2:** Passive monitoring method using personal concentration samplers for O_3 and NO_2.	***Oxidative stress:*** *Superoxide dismutase (SOD).*<br>***Reactive oxygen species (ROS):*** *Fluorescence intensity using the redox-sensitive dye 2′,7′-dichlorodihydrofluorescein diacetate (DCFH, 100 μM, Sigma-Aldrich).*<br>***Catalase:*** *Measured with a Lambda 35 spectrophotometer (PerkinElmer Brazil, São Paulo, Brazil).*<br>***Glutathione (GSH):*** *Quantified using the QuantiChrom™ Glutathione Assay Kit (BioAssay Systems, CA, USA).*<br>***Interleukins (IL):*** *IL-1β (BD Biosciences, USA), IL-4, IL-6, IL-10, TNF-α (Thermo Fisher, USA), CC16 (ElabScience, USA), and HSP70 (Enzo Biotech, USA) were quantified using an ELISA microplate reader (SpectraMax M2/M2e Microplate Reader – Molecular Devices, USA).*

BC = Black carbon, **NO_2** = Nitrogen dioxide, **UP** = Ultrafine particles, **PM** = Particulate matter, **T** = Temperature, **RH** = Relative humidity, **MRC** = Medical Research Council, **HR** = Heart rate, **FeNO** = Fractional exhaled nitric oxide, **FEV_1** = Forced expiratory volume in 1 second, **FVC** = Forced vital capacity, **PEF** = Peak expiratory flow, **NO** = Nitric oxide, **NO_x** = Nitrogen oxides, **SO_2** = Sulfur dioxide, **O_3** = Ozone, **PNC** = Particle number concentration, **HRmax** = Maximum heart rate, **IL** = Interleukins, **TNF** = Tumor necrosis factor, **ROS** = Reactive oxygen species
Source: Authors' own elaboration.
